# Platelet Reactivity and Outcomes after Off-Pump Coronary Surgery in Acute Coronary Syndrome Patients

**DOI:** 10.3390/jcm11123285

**Published:** 2022-06-08

**Authors:** Sarah Soh, Yu Rim Shin, Jong-Wook Song, Jun Hyug Choi, Young-Lan Kwak, Jae-Kwang Shim

**Affiliations:** 1 Department of Anesthesiology and Pain Medicine, Yonsei University College of Medicine, 50-1 Yonsei-ro, Seodaemun-Gu, Seoul 03722, Korea; yeonchoo@yuhs.ac (S.S.); sjw72331@yuhs.ac (J.-W.S.); cjh0208@yuhs.ac (J.H.C.); ylkwak@yuhs.ac (Y.-L.K.); 2 Anesthesia and Pain Research Institute, Yonsei University College of Medicine, 50-1 Yonsei-ro, Seodaemun-Gu, Seoul 03722, Korea; 3 Division of Cardiovascular Surgery, Department of Thoracic and Cardiovascular Surgery, Yonsei University College of Medicine, 50-1 Yonsei-ro, Seodaemun-Gu, Seoul 03722, Korea; yull0629@yuhs.ac

**Keywords:** acute coronary syndrome, dual anti-platelet therapy, off-pump coronary artery bypass surgery, P2Y_12_ antagonist

## Abstract

Ischemic and hemorrhagic complications are major determinants of survival in acute coronary syndrome (ACS) patients undergoing coronary surgery. We investigated the association of preoperative platelet reactivity to P2Y_12_ antagonists with ischemic and hemorrhagic complications after Off-Pump Coronary Artery Bypass surgery (OPCAB) in ACS patients who received dual anti-platelet therapy (DAPT) within 5 days prior to surgery. This prospective, observational study with 177 patients compared the incidence of perioperative major bleeding and major adverse cardiac events (MACEs) in relation to the tertile distribution of the % inhibitory response to P2Y_12_ antagonists, as measured by a thromboelastography platelet mapping assay. The incidences of perioperative major bleeding and MACEs were similar in relation to the tertile distribution of inhibitory response to P2Y_12_ antagonists. The % inhibitory responses to P2Y_12_ antagonists between patients who did or did not exhibit MACEs, and with or without major bleeding, were 58 ± 20% and 56 ± 20% (*p* = 0.578) and 57 ± 19% and 56 ± 21% (*p* = 0.923), respectively. In ACS patients who received DAPT close to OPCAB, the platelet inhibitory response to P2Y_12_ antagonists was not associated with ischemic or hemorrhagic complications. OPCAB may obviate the need for routine platelet function testing for ACS patients requiring DAPT and surgical revascularization. Clinical Registration Number: NCT02184884.

## 1. Introduction

Dual anti-platelet therapy (DAPT) has been used for prophylaxis against recurrent ischemic attacks in acute coronary syndromes (ACS) [1]. This challenges the guidelines recommending P2Y_12_ antagonist discontinuation before surgery due to heightened bleeding risks [2]. Both ischemic and bleeding complications are major determinants of survival in ACS patients undergoing coronary bypass graft surgery (CABG) [3]. Unfortunately, the ischemic and hemorrhagic responses to DAPT vary among individuals and cannot be predicted by clinical risk factors alone.

Platelet function tests play a valuable role in predicting clinical outcomes in ACS patients undergoing percutaneous coronary intervention (PCI) [4]. A collaborative analysis involving 20,839 PCI patients reported that there may be an optimal therapeutic window of P2Y_12_ inhibition, within which the anticipated risks of ischemic and bleeding complications are the lowest [5].

Thus, we hypothesized that a similar optimal therapeutic window is applicable to ACS patients undergoing CABG, providing a safe perioperative milieu with regard to ischemic and bleeding complications, and eliminating the need for unnecessary delay from DAPT interruption. However, evidence regarding the role of platelet function tests in CABG is scarce and is mostly limited to its negative predictive value, indicating that surgery may proceed when there is low platelet inhibition [6,7]. Such evidence is even more scarce in ACS patients. For patients on DAPT, off-pump CABG (OPCAB) has been suggested as a rational choice for surgical revascularization, as it evades cardiopulmonary bypass (CPB)-related coagulopathy [8].

This prospective, observational study aimed to investigate the association of preoperative platelet reactivity to adenosine diphosphate (ADP), as assessed by thromboelastography platelet mapping, with ischemic and bleeding complications in ACS patients who received DAPT within 5 days prior to surgery.

## 2. Materials and Methods

### 2.1. Patients

**Figure 1:**
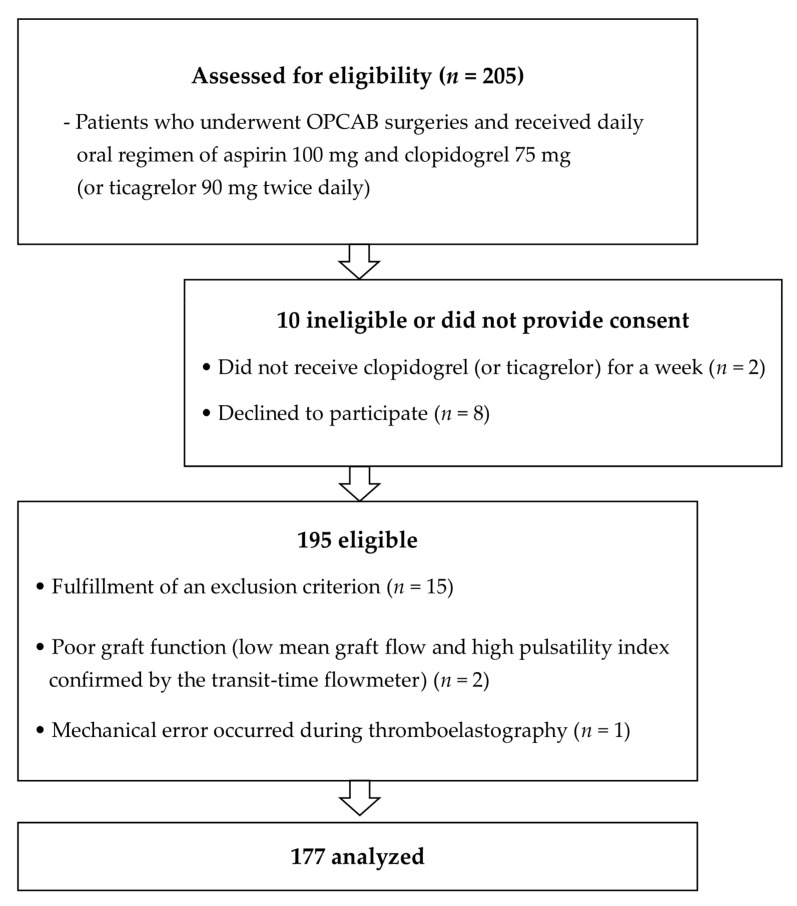
Patient enrolment into the study. The figure illustrates the inclusion and exclusion criteria applied in the study and the resulting number of patients that were finally analyzed. OPCAB, off-pump coronary surgery.

A total of 205 patients who underwent multivessel OPCAB between June 2014 and December 2019 and who were taking a daily oral regimen of aspirin 100 mg and clopidogrel 75 mg (or 90 mg of ticagrelor twice daily) for at least 1 week were assessed for eligibility. Patients who discontinued DAPT for more than 5 days pre-surgery (3 days for ticagrelor) were excluded. Exclusion criteria included emergency surgery, bleeding diathesis or hepatic dysfunction, left ventricular ejection fraction < 40%, hemoglobin < 11 g/dL, platelet count < 100,000/μL, creatinine > 1.4 mg/dL, and glycoprotein IIb/IIIa inhibitor or anticoagulants use. Patients with poor graft patency, as confirmed intraoperatively using a transit time flow measurement after completion of grafting, were also excluded. We finally included 177 patients. (Figure 1).

### 2.2. Primary Endpoint

The primary endpoint was a comparison of the incidence of perioperative major adverse cardiovascular events (MACEs) and major bleeding in relation to the tertile distribution of the % platelet inhibitory response to P2Y_12_ antagonists.

A thromboelastography platelet mapping assay was used to measure the platelet reactivity. Major bleeding was defined as class ≥2 (moderate) universal definition of perioperative bleeding (UDPB), which is as follows: delayed sternal closure, postoperative bleeding >800 mL within 12 h, transfusion of ≥2 units of packed erythrocytes (pRBCs), fresh frozen plasma (FFP), platelets or cryoprecipitate transfusion, use of prothrombin complex concentrates or recombinant activated factor VII, or the need for re-exploration [9]. MACEs were defined as in-hospital cardiac death, myocardial infarction, or need for revascularization. Postoperative myocardial infarction was defined according to the universal definition [10]. Troponin-T was evaluated 1-day pre-surgery and at 24-h and 48-h post-surgery.

### 2.3. Secondary Endpoints

MACEs and major bleeding risk factors, including the % platelet inhibitory response to antiplatelet agents, were evaluated. Additionally, we evaluated transfusion requirements and other major morbidity endpoints. These endpoints included postoperative stroke, acute kidney injury, prolonged ventilation >24 h, deep sternal wound infection, any cardiac reoperation, and pulmonary thromboembolism. Acute kidney injury was diagnosed according to the Kidney Disease: Improving Global Outcomes criteria [11]. Computerized tomography coronary angiography was performed within 2 weeks post-CABG to assess the graft patency and pulmonary thromboembolism occurrence. Other complications were assessed according to the Society of Thoracic Surgeons’ morbidity definition. (https://www.sts.org/quality-safety/performance-measures/descriptions, accessed on 6 April 2014)

### 2.4. Data Collection

Blood sampling and a thromboelastography^®^ Platelet Mapping™ assay (Haemoscope Corp., Niles, IL, USA) were performed immediately before anesthesia induction [6] by an anesthesiologist not involved in the patient management (S.S). The surgeons and anesthesiologists involved in patient management were blinded to the results.

The perioperative data assessed were patient characteristics, operation time, need for proximal aortocoronary anastomosis, the number of grafts placed, blood loss within the first 24 h post-surgery (intra- and post-operative blood loss), and transfusion requirements for pRBCs, FFP, and platelets during the same period. Perioperative laboratory data, including hemoglobin, platelet counts, prothrombin time, activated partial thromboplastin time, and troponin T were evaluated. The lengths of hospital and intensive care unit stays were also recorded.

### 2.5. Clinical Management

Institutional standard anesthetic and surgical management and intensive unit care were provided to all patients, as in our previous study [6]. Routine monitoring included pulmonary artery catheter and transesophageal echocardiography. Anesthesia was maintained with sevoflurane and sufentanil. All surgical procedures were performed via median sternotomy by two teams of cardiac surgeons, who each had more than 500 cases of OPCAB surgeries before the study. The target activated clotting time for systemic heparinization was 250–300 s. Proximal aortocoronary anastomosis was performed with the Heartstring device (Maquet Cardiovascular, San Jose, CA, USA). A cell-salvage device was used in all patients and intraoperative blood loss was estimated by the amount of blood collected by it. Salvaged blood was transfused to the patient before leaving the operating theatre. No patient received antifibrinolytics, and pRBCs were transfused at hemoglobin levels < 8 g/dL at the discretion of the attending physician.

When bleeding exceeded 200 mL/h for 2 consecutive hours post-surgery, FFP and/or platelets were transfused, in case of an international normalized ratio >1.3 and/or platelet counts < 50,000/μL. Reoperation was performed when bleeding exceeded 200 mL/h for ≥6 h or ≥400 mL for the first 1 h. In laboratory tests, if there was an increase in the international normalized ratio or a decrease in the platelet count that did not meet the transfusion criteria, and the bleeding exceeded 200 mL/h for 3 h, FFP or platelet transfusion was decided at the discretion of the attending physician. DAPT was restarted within 24 h post-surgery in the absence of major bleeding.

### 2.6. Statistical Analyses

SPSS v25.0 (SPSS Inc., Chicago, IL, USA) was used for statistical analysis. In patients with ACS, the incidence of perioperative MACEs was assumed to be 20% (unpublished institutional data). We determined that estimating a 10% dropout rate, 195 patients would be required to detect a two-fold increase in the incidence of MACEs in the first tertile of P2Y_12_ receptor antagonist responsiveness (showing the lowest percentage of platelet inhibition) at α = 0.05 and β = 0.8.

Data are shown as mean (SD), median (IQR), or number of patients (percentage). Normality was assessed using the Shapiro–Wilk test. Continuous variables were compared among the tertiles by one-way analysis of variance with Bonferroni post-hoc tests. Continuous variables were compared between patients with and without postoperative MACEs or major bleeding (UDPB ≥ class 2) with the independent *t*-test, or Mann–Whitney U-test. Proportions were compared using Fisher’s exact test or χ^2^ test. Multivariable logistic regression analysis was used to determine if the mean % platelet inhibitory response to P2Y_12_ antagonists or aspirin was an independent risk factor for major bleeding. Known risk factors of UDPB (EuroSCORE, preoperative hemoglobin, and number of grafts (instead of CPB duration)) [9] and the mean % platelet inhibitory response to P2Y_12_ antagonists or aspirin were included in the multivariable analysis. Odds ratios (OR) and associated 95% confidence intervals were calculated. *p* < 0.05 was considered statistically significant.

## 3. Results

OPCAB was completed in all 177 patients (95% of patients received clopidogrel, 5% received ticagrelor). Of the patients in the study, 29 (16%) were diagnosed with non-ST-elevation myocardial infarction preoperatively, and the rest were unstable angina. Among them, 40% and 75% of patients took P2Y_12_ antagonists and aspirin, respectively, until the day before surgery. Patients’ characteristics, including the number of patients who stopped P2Y_12_ antagonists and/or aspirin 1 day before surgery, were all similar in terms of tertile distributions of % platelet inhibitory response to P2Y_12_ antagonists, except for the % platelet inhibitory response to aspirin (Supplementary Table S1). The mean % platelet inhibitory responses to ADP and arachidonic acid were 56% (20%) and 73% (21%), respectively.

The perioperative blood loss and transfusion requirements were all similar according to the tertile distribution of the % platelet inhibitory response to P2Y_12_ antagonists, except for the platelet concentrate transfusion (Table 1). Significantly more patients in the third tertile required platelet concentrate transfusion.

The MACEs and major bleeding incidences were 17% and 40%, respectively. The incidence of MACEs and other morbidity endpoints was similar among the tertiles, with the exception of acute kidney injury, which was significantly higher in patients in the second tertile than in those in the first and third tertiles (Table 2). In linear regression analysis, the % platelet inhibitory response to P2Y_12_ antagonists before OPCAB and peak troponin-T values for 48 h postoperatively showed no significant correlation (R2 = 0.003, *p* = 0.5).

None of the thromboelastography parameters, including the mean % platelet inhibitory response to P2Y_12_ antagonists, were associated with MACEs or major bleeding (UDPB ≥ class 2) (Table 3). The % platelet inhibitory response to P2Y_12_ antagonists and aspirin showed no correlation with the amount of postoperative bleeding within 12 h (R = 0.06 and 0.115, *p* = 0.43 and 0.13, respectively) or 24 h (R = 0.05 and 0.123, *p* = 0.5 and 0.11, respectively). Even when a partial correlation analysis was used to adjust the confounding effect of platelet transfusion, there was no significant correlation between the % platelet inhibitory response to antiplatelet therapy (P2Y_12_ antagonists and aspirin) and the amount of postoperative bleeding within 12 h (R = 0.03 and 0.075, *p* = 0.69 and 0.33, respectively) or 24 h (R = 0.033 and 0.1, *p* = 0.66 and 0.19, respectively).

Logistic regression analysis to identify major bleeding risk factors concomitantly analyzed the previously cited risk factors and the variable of interest, % inhibitory response to P2Y_12_ receptor antagonists. However, none of these variables remained as independent risk factors in the multivariable analysis (Table 4). Compared with a previous study [6], the results did not change even when the % inhibitory response to P2Y_12_ antagonist was added as a dichotomous variable with a cut-off value of 70% (Supplementary Table S2).

## 4. Discussion

This prospective, observational study demonstrated that in ACS patients with recent exposure to DAPT pre-OPCAB, the % platelet inhibitory response to P2Y_12_ antagonists was not associated with increased incidence of perioperative ischemic or hemorrhagic complications.

The existence of on-treatment response variability as well as variability in platelet function recovery after discontinuation of P2Y_12_ antagonists stands against the uniform cessation-protocol and suggests the need for objective measurement of platelet inhibitory response to P2Y_12_ antagonists [4]. Recent studies have suggested the existence of a therapeutic window of platelet reactivity in patients undergoing PCI treated with P2Y_12_ antagonists where maximum ischemic benefits can be achieved while avoiding excessive bleeding [4]. Such therapeutic windows would be of particular clinical relevance to ACS patients undergoing CABG, as these patients stand at high risk of both ischemic and bleeding complications while confronting the need for discontinuation of P2Y_12_ inhibitors [12,13]. Studies of patients undergoing CABG showed that an individualized strategy for preoperative clopidogrel cessation, based on preoperative platelet function tests, could reduce both preoperative delay and bleeding diatheses [6,14]. Current guidelines issue Class IIa or IIb recommendations for preoperative platelet function tests [15,16]. However, most evidence stems from on-pump CABG requiring CPB, which inevitably accompanies hemodilution and consumptive coagulopathy.

In that context, OPCAB is an important alternative surgical technique to reduce hemorrhagic complications related to recent P2Y_12_ antagonist exposure [8,17]. OPCAB may allow surgical revascularization in ACS patients requiring DAPT and could provide ischemic protection without markedly increasing bleeding risk; however, no comprehensive evidence exists. Thus, we investigated the association between the degree of preoperative P2Y_12_ inhibition and the occurrence of bleeding as well as ischemic complications in ACS patients undergoing OPCAB.

Contrary to expectations, platelet inhibition levels measured using thromboelastography platelet mapping before OPCAB were not associated with the incidence of perioperative MACEs. Moreover, preoperative baseline troponin-T levels and serially assessed postoperative troponin-T levels did not correlate with the degree of platelet inhibition. Additionally, the incidence of postoperative stroke, pulmonary thromboembolism, and cardiac death was relatively low, and those events did not correlate with the degree of platelet inhibitory response to P2Y_12_ antagonists.

Cogent explanations for the observed results are as follows. DAPT maintenance for up to 5 days prior to OPCAB in all patients (82% of patients took P2Y_12_ antagonists within 3 days prior to surgery) might have exerted ischemic benefits, not only during the preoperative waiting period but also against the perioperative occurrence of MACEs. Such a benefit could have been strengthened further by the “East Asian paradox” [18], where rates of ischemic events are lower with clopidogrel use despite a higher rate of high on-treatment platelet reactivity.

Furthermore, unlike the accumulating evidence of an association between the extent of platelet inhibition and CABG-related bleeding [6,19,20], the degree of platelet inhibition in ACS patients undergoing OPCAB was not associated with postoperative major bleeding occurrence. Despite inclusion of only patients with ACS in this study, putative reasons for discrepancy are as follows. First, the increased bleeding risk in patients with ACS is attributable to the more aggressive DAPT, rather than the ACS per se [21]. Patients enrolled in the current study did not have major risk factors of bleeding other than receiving DAPT; only 9 patients (5%) met the criteria for severe (class 3) postoperative bleeding in the current study, as opposed to 8.2%, according to the UDPB [9], while none of the patients exhibited massive bleeding (class 4 UDPB: >2000 mL blood loss within 12 h). Additionally, all patients underwent OPCAB, which could have reduced the risk of bleeding by avoiding CPB. Even if the focus is on P2Y_12_ antagonists, the relatively lower efficacy of P2Y_12_ antagonists could have affected our results. In a previous study, using the same thromboelastography platelet mapping assay, patients exhibiting the highest % inhibitory response to clopidogrel (third tertile) had significantly more postoperative bleeding than those in the first and second tertiles [6]. The mean % inhibitory response to P2Y_12_ antagonists in the current study’s third tertile was comparatively lower (79% vs. 87%) [6]. Therefore, in patients at low risk of postoperative bleeding, other than those receiving DAPT, there seems to be no need to postpone OPCAB based on the degree of platelet inhibitory response to P2Y_12_ antagonists. Nonetheless, as the cause of bleeding after CABG is multifactorial, it is difficult to deduce the role of platelet function testing in patients with other major risk factors of bleeding.

Overall, our findings imply a ceiling-effect of ischemic protection against MACEs by DAPT at a low degree of P2Y_12_ receptor inhibition (in the presence of aspirin continuation, the mean percent inhibitory response to ADP and arachidonic acid were 56% and 73%, respectively), without inciting serious bleeding complications. Moreover, the current study adds clinical significance as the majority (82%) of patients received P2Y_12_ antagonists within 3 days prior to surgery (40% of patients until 1 day prior) and not 4 or 5 days prior. Thus, our results strongly imply ischemic and hemorrhagic benefits of OPCAB in ACS patients who received DAPT in proximity to surgery.

This study is subject to the following limitations. First, this is a single-center study of a certain ethnic background with specific inclusion criteria, which limits generalization of the observed results. Secondly, the physicians were not blinded to DAPT exposure, which may have confounded decisions regarding platelet transfusions and the subsequent amount of blood loss. However, as in a previous study [6], the distribution of discontinuation dates was similar in relation to tertile distributions of the % inhibitory response to ADP in this study, reflecting the inter-individual variability of the drug response when the discontinuation dates of the P2Y_12_ inhibitor were mostly within a close range of 1 and 3 days before surgery (Supplementary Table S1). In addition, the degree of P2Y_12_ inhibition showed no correlation with the amount of postoperative bleeding even after adjustment for platelet transfusion. Thirdly, discontinuation of P2Y_12_ antagonists before surgery was at the discretion of the attending cardiologists and surgeons.

In conclusion, in ACS patients who continued to receive DAPT up to 5 days prior to surgery, the degree of platelet inhibitory response to P2Y_12_ antagonists before OPCAB was not associated with perioperative ischemic or hemorrhagic complications. Considering the low incidences of serious ischemic and bleeding complications in the current study, our results imply that routine platelet function testing may not be necessary to identify the optimal timing of surgery for ACS patients requiring DAPT, whose risk of bleeding is not increased, when undergoing planned OPCAB.

## Figures and Tables

**Table 1:** Perioperative data in relation to tertile distribution of percentage of platelet inhibitory response to adenosine diphosphate.

	Total (*n* = 177)	First Tertile (3–47%)	Second Tertile (48–66%)	Third Tertile (66–99%)	*p*-Value
Operation time, min	239 ± 38	237 ± 37	234 ± 41	245 ± 35	0.231
No. of grafts performed	4 (3–4)	3 (3–4)	4 (3–4)	4 (3–4)	0.32
Proximal AC anastomosis, *n*	114 (64%)	41 (70%)	38 (64%)	35 (59%)	0.514
Blood loss, mL					
Intraoperative	697 (500–900)	660 (450–900)	650 (460–800)	700 (550–1000)	0.324
Postoperative 12 h	470 (342–624)	430 (329–580)	480 (352–653)	513 (405–630)	0.284
Perioperative transfusion (up to 12 h after surgery)					
No. of patients transfused with pRBCs	49 (28%)	15 (25%)	18 (31%)	16 (27%)	0.821
pRBCs transfusion, U, median (5th percentile-95th percentile)	0 (0–2.1)	0 (0–2)	0 (0–3)	0 (0–2)	0.709
No. of patients transfused with FFP	53 (30%)	16 (27%)	21 (36%)	16 (27%)	0.51
FFP transfusion, U, median (5th percentile-95th percentile)	0 (0–4)	0 (0–5)	0 (0–3)	0 (0–5)	0.681
No. of patients transfused with platelet concentrate	25 (14%)	5 (9%)	6 (10%)	14 (24%)	0.033
Platelet concentrate transfusion, U, median (5th percentile-95th percentile)	0 (0–6)	0 (0–6)	0 (0–6)	0 (0–12)	0.035
Postoperative laboratory data					
Postoperative day 0					
Hemoglobin, g/dL	9.8 ± 1.5	9.8 ± 1.4	9.9 ± 1.8	9.9 ± 1.4	0.952
Platelet count, 10^3^/mm^2^	151 (132–181)	157 (137–184)	149 (125–176)	150 (128–183)	0.474
Prothrombin time, s	12.8 (12.2–13.5)	12.5 (12–13.5)	12.9 (12.3–13.5)	12.9 (12.2–13.8)	0.371
aPTT, s	31.0 (28.8–33.7)	31.5 (29.2–34.2)	30.8 (28.8–37.8)	30.8 (28.3–33.1)	0.274
Troponin T, pg/mL	112 (77–169)	104 (71–170)	112 (78–197)	118 (81–155)	0.301
Postoperative day 1					
Hemoglobin, g/dL	9.5 ± 1.3	9.4 ± 1.2	9.5 ± 1.3	9.6 ± 1.3	0.791
Platelet count, 10^3^/mm^2^	140 (120–183)	143 (124–184)	135 (113–161)	144 (125–188)	0.243
Prothrombin time, s	13.4 (12.7–14.2)	13.2 (12.6–14.4)	13.5 (12.7–14)	13.4 (12.8–14.2)	0.785
aPTT, s	31.9 (29.3–36.9)	32.3 (28.9–39.7)	32.1 (29.7–36.6)	31.5 (29.2–35.5)	0.802
Troponin T, pg/mL	116 (94–184)	104 (78–168)	136 (96–214)	116 (94–171)	0.031

Data are presented as mean ± standard deviation (SD), median (interquartile range, IQR), or number of patients (%). AC, aortocoronary; aPTT, activated partial thromboplastin time; FFP, fresh frozen plasma; pRBCs, packed erythrocytes.

**Table 2:** Changes in troponin T values and postoperative outcomes.

	Total (*n* = 177)	First Tertile (3–47%)	Second Tertile (48–66%)	Third Tertile (66–99%)	*p*-Value
Nonfatal myocardial infarction	29 (16%)	6 (10%)	14 (24%)	9 (15%)	0.133
Peak troponin-T levels 48 h after surgery, pg/mL	143 (109–218)	126 (96–199)	167 (120–262)	147 (117–206)	0.098
Stroke	1 (0.6%)	0 (0%)	0 (0%)	1 (2%)	>0.999
Acute kidney injury (KDIGO criteria)	32 (18%)	11 (19%)	16 (27%)	5 (9%)	0.031
Prolonged ventilation, >24 h	12 (7%)	4 (7%)	2 (3%)	6 (10%)	0.399
Deep sternal wound infection	8 (5%)	2 (3%)	4 (7%)	2 (3%)	0.733
Re-operation	2 (1%)	1 (2%)	0 (0%)	1 (2%)	>0.999
Bleeding categories according to the UDPB (≥Class 2)	70 (40%)	20 (34%)	26 (44%)	24 (41%)	0.516
Class 2 (moderate)	61 (35%)	17 (29%)	25 (42%)	19 (32%)	0.308
Class 3 (severe)	9 (5%)	3 (5%)	1 (2%)	5 (9%)	
Pulmonary thromboembolism	8 (4%)	4 (7%)	1 (2%)	3 (5%)	0.537
Length of stay in the ICU, days	2 (2–3)	2 (2–3)	2 (2–2)	2 (2–3)	0.258
Length of postoperative hospitalization, days	11 (9–14)	11 (9–14)	11 (9–13)	11 (10–14)	0.512
30-day cardiac mortality after surgery	2 (1%)	1 (2%)	1 (2%)	0 (0%)	>0.999
Major adverse cardiovascular events	30 (17%)	7 (12%)	14 (24%)	9 (15%)	0.209

Table depicts the changes in troponin T values and postoperative outcomes in relation to tertile distribution of percentage of platelet inhibitory response to adenosine diphosphate. Data are presented as median (interquartile range, IQR), or number of patients (%). ICU, intensive care unit; KDIGO, Kidney Disease: Improving Global Outcomes; UDPB, universal definition for perioperative bleeding.

**Table 3:** Preoperative thromboelastography parameters.

	**Total** **(*n* = 177)**	**MACE** **(*n* = 30)**	**No MACE** **(*n* = 147)**	***p*-Value**
Preoperative thromboelastography parameters				
% inhibitory response to ADP	56 ± 20	58 ± 20	56 ± 20	0.578
% inhibitory response to aspirin	73 ± 21	77 ± 19	72 ± 21	0.272
Maximal amplitude, mm	61 ± 6	60 ± 7	61 ± 6	0.287
	**Total** **(*n* = 177)**	**UDPB Class 2–3** **(*n* = 70)**	**UDPB Class 0–1** **(*n* = 107)**	***p*-Value**
Preoperative thromboelastography parameters				
% Inhibitory response to ADP	56 ± 20	57 ± 19	56 ± 21	0.923
% Inhibitory response to aspirin	73 ± 21	77 ± 21	71 ± 20	0.064
Maximal amplitude, mm	61 ± 6	60 ± 5	61 ± 6	0.576

Preoperative thromboelastography parameters according to the occurrence of major adverse cardiac events or major bleeding complications. Data are presented as mean ± standard deviation (SD). ADP, adenosine diphosphate; MACE, major adverse cardiovascular event; UDPB, universal definition of perioperative bleeding.

**Table 4:** Predictive power of chosen variables.

Variables	Multivariable Analysis	
	Odds Ratio (95% CI)	*p*-Value
EuroSCORE (logistic)	0.914 (0.818–1.022)	0.113
No. of grafts performed	1.279 (0.885–1.849)	0.19
Preoperative hemoglobin	0.875 (0.686–1.114)	0.278
Thromboelastography parameters		
% inhibitory response to P2Y_12_ inhibitors	0.994 (0.976–1.012)	0.493
% inhibitory response to aspirin	1.016 (0.997–1.035)	0.095

Predictive power of chosen variables for perioperative major bleeding (UDPB ≥ class 2) according to logistic regression analyses. UDPB, universal definition for perioperative bleeding; CI, confidence interval.

## Data Availability

The datasets used or analyzed during the current study are available from the corresponding author on reasonable request.

## Supplementary Materials

The following supporting information can be downloaded at: https://www.mdpi.com/article/10.3390/jcm11123285/s1, Table S1: Patient characteristics in relation to tertile distribution of the platelet inhibitory percentage response to adenosine diphosphate; Table S2: Predictive power of chosen variables.
