# Supplementary material for: Platelet Reactivity and Outcomes after Off-Pump Coronary Surgery in Acute Coronary Syndrome Patients
Source: J Clin Med. 2022 Jun 8;11(12):3285. doi: 10.3390/jcm11123285 (PMC9224657; doi:10.3390/jcm11123285)
Supplement: Supplementary file 1 [file jcm-11-03285-s001.zip › jcm-1739371-supplementary.pdf]

Supplementary Table S1. Patient characteristics in relation to tertile distribution of the platelet inhibitory percentage response to adenosine diphosphate

	Total (n=177)	First tertile (3%–47%)	Second tertile (48%–66%)	Third tertile (66%–99%)	p-value
Age, years	65 ± 9	67 ± 10	65 ± 10	63 ± 9	0.108
Females	37 (21%)	17 (29%)	13 (22%)	7 (12%)	0.075
Body mass index, kg/m^2	25.1 ± 3.2	25.6 ± 3.2	24.6 ± 3.1	24.9 ± 3.3	0.228
Pre-operative medication					
Beta-blocker	104 (59%)	40 (68%)	35 (59%)	29 (49%)	0.12
RAS antagonists	91 (51%)	29 (49%)	34 (58%)	28 (48%)	0.496
Calcium channel blocker	78 (44%)	29 (49%)	27 (46%)	22 (37%)	0.409
Statins	143 (81%)	46 (78%)	46 (78%)	51 (86%)	0.402
Hypertension	119 (67%)	42 (71%)	41 (70%)	36 (61%)	0.452
Diabetes mellitus	84 (48%)	31 (53%)	24 (41%)	29 (49%)	0.413
Left ventricular ejection fraction, %	60 (50-69)	63 (50-69)	60 (51-69)	59 (49-67)	0.639
Left main coronary artery disease	44 (26%)	16 (28%)	14 (26%)	14 (24%)	0.887
$P2Y_{12}$ antagonist discontinued date					0.608
1 day before surgery	72 (41%)	25 (42%)	22 (37%)	25 (42%)	
2-3 days before surgery	73 (41%)	27 (46%)	24 (41%)	22 (37%)	
4-5 days before surgery	32 (18%)	7 (12%)	13 (22%)	12 (20%)	
Aspirin stopped 1 day before surgery	133 (75%)	44 (75%)	39 (66%)	50 (85%)	0.064
EuroSCORE (logistic, %)	3.4 (2.3–5.7)	3.9 (2.3–6.8)	3.4 (2.3–4.4)	3.2 (2.1–5)	0.184
NYHA III, IV	21 (12%)	7 (12%)	7 (12%)	7 (12%)	0.999
Thromboelastography parameters					

% inhibitory response to ADP	56 ± 20	35 ± 12	56 ± 6	79 ± 9	<0.001
% inhibitory response to aspirin	73 ± 21	62 ± 23	70 ± 18	87 ± 11	<0.001
Maximal amplitude, mm	61 ± 6	61 ± 6	61 ± 6	60 ± 6	0.294
Laboratory data					
Creatinine, mg/dl	0.84 (0.74–0.98)	0.83 (0.68–0.91)	0.85 (0.75–0.99)	0.87 (0.75–0.99)	0.335
Hemoglobin, g/dl	13.6 ± 1.4	13.6 ± 1.5	13.5 ± 1.3	13.8 ± 1.5	0.492
Platelet count, $10^3/mm^2$	208 (177–252)	219 (187–262)	201 (177–238)	208 (172–254)	0.291
Prothrombin time, s	11.1 (10.6–11.7)	11 (10.6–11.5)	11.1 (10.5–11.9)	11.2 (10.7–11.7)	0.583
aPTT, s	35.1 (30.1-52.8)	36.4 (30.9-54.5)	33.5 (29.3-49)	35 (30-54.6)	0.357
Antithrombin III, %	87 (81–95)	86 (81–96)	88 (83–95)	87 (81–94)	0.598
Fibrinogen, mg/dl	325 ± 55	320 ± 51	327 ± 58	327 ± 57	0.754
LDL cholesterol, mg/dl	78 (59–96)	81 (62–101)	77 (61–102)	73 (55–90)	0.351
Troponin T, pg/ml	12 (8–21)	12 (7–18)	16 (8–44)	10 (8–15)	0.119

Data are presented as mean ± standard deviation (SD), median (interquartile range, IQR), or number of patients (%)

RAS, renin–angiotensin system; NYHA, New York Heart Association functional classification; aPTT, activated partial thromboplastin time; LDL, low-density lipoprotein.

Supplementary Table S2. Predictive power of chosen variables.

Predictive power of chosen variables for perioperative major bleeding (UDPB ≥class 2) according to logistic regression analyses

Variables	Multivariable analysis	
	Odds ratio (95% CI)	p–value
EuroSCORE (logistic)	0.915 (0.819–1.022)	0.114
No. of grafts performed	1.274 (0.882–1.84)	0.197
Preoperative hemoglobin	0.871 (0.685–1.109)	0.264
Thromboelastography parameters		
% inhibitory response to $P2Y_{12}$ inhibitors >70%	0.791 (0.359–1.743)	0.561
% inhibitory response to aspirin	1.015 (0.997–1.033)	0.102

UDPB, universal definition for perioperative bleeding; CI, confidence interval; EuroSCORE, European System for Cardiac Operative Risk Evaluation
